# A Case of Placenta Prævia, Terminating in Death on the Thirteenth Day after Delivery

**Published:** 1872-05

**Authors:** W. F. Hutchinson

**Affiliations:** Minneapolis, Minn.


ART. II.—*A case of Placenta Prævia, terminating in death on the thirteenth day after delivery. By* W. F. HUTCHINSON, M. D. *Minneapolis, Minn.*

Tuesday, Feb. 1, 1872.—I was called to attend the accouchement of Mrs. G. aet. 40, primapara.

For two or three days previously a slight discharge of blood had been noticed, which had gradually increased in quantity until suspicion was aroused that something beyond the usual phenomena of a lying-in chamber was about to occur.

I found the lady comfortable, except that the flow of blood was greater than it had been, and that she felt slight premonitory pains. When making an examination by the touch I discovered a remarkable degree of ante-version; so great that the os uteri was too far up in the hollow of the sacrum to be reached by the finger. I therefore slowly and cautiously introduced the entire hand, and found the labor begun, with a dilatation of about one half inch. The presentation could be made out as vertex, and the edge of the placenta felt, distinguished by its peculiar textile feel, implanted on the left side of the cervix. At this time she was bleeding but not to any great extent, active dilatation not yet having commenced.

As there was plenty of time, counsel was summoned and the diagnosis verified. The os was tense, hard and thin, and, with the slightness of the bleeding and great strength of the patient it was decided to wait.

The vagina was carefully tamponed, a quarter of a grain of morphia administered, and the case left for a few hours rest, under the supervision of Mr. Neill, medical student.

Eight P. M. Patient had slept quietly and Mr. Neill reported only slight oozing, apparently between the tampon and maternal organs. Pulse 80, full and regular.

The tampon was cautiously removed and dilatation found to be increased to about two and a half inches,. with the os in the same position, viz: the hollow of the sacrum, looking backwards, tense and hard. Pains now begun to recur at intervals of from fifteen to twenty minutes, a gush of blood accompanying each contraction.

The membranes were ruptured, a drachm of Squibbs' fluid extract of ergot administered, and all preparations made for version, but in a few moments, the pains became stronger, and the head descending, impinged upon the placenta, shutting off the blood.

From this time labor progressed steadily until at three A. M., the child was born, accompanied with so great a gush of blood as to excite fear of immediate dissolution. Introducing the hand, the placenta was at once detached from its uterine attachment and thrust into the vagina, the hand being retained in the uterine cavity. Contraction was induced by firm pressure externally with iced cloths, and titillation within, administering at the same time two drachms more of the ergot in an ounce of brandy. In ten minutes the organ threw out the hand and was reduced to about the size of the fœtal head, with srarcely a trace of the previous frightful hemorrhage. The child, a strong female, was then severed from the placenta and attended to.

Patient expressed herself comfortable, but was very weak and exhausted, with a small fluttering pulse at 90, and great tendency to faintness. She was enjoined strict quiet, the hips raised, and brandy and carbonate of ammonia freely given until she revived, when the child was applied to the breast to further excite the uterus to contraction.

At 6 A. M., all being quiet, a firm compress was applied over the hypogastruim and the patient left to rest.

Called at 9 A. M., and found my patient still quiet, but very weak, Pulse unchanged.

On inspection of the pudenda, I discovered large clots of blood, and introducing the finger into the vagina, found it filled with the same. These were turned out, iced cloths applied to the vulva, and a mixture of aromatic sulphuric acid, ergot and opium directed to be given every second hour. The bleeding being thereby again checked. she was placed upon a diet of cold beef essence and Fougera's Nutritive Wine, a preparation said to contain an ounce of the soluble constituents of beef to each ounce of wine.

From this time for five days no untoward symptom appeared, all going on nicely. Upon the morning of the sixth, I was summoned with great haste. She was bleeding again. When arriving at the house, two miles distant, I found her lying in a pool of blood, apparently moribund.

The uterus had completely relaxed, so as to admit the hand with ease still retaining its extreme anteversion. Ice, ergot and stimulants were freely employed, with the effect of again arresting the flow, and brandy milk punch ordered in place of the nutritive wine.

I regret to be compelled to state that from this time to her death which occurred seven days thereafter, all treatment was useless, and Mrs. G. presented the singular spectacle of slow death by inanition, while the appetite was good, the mind unclouded and the amount of ingesta averaged a pound of beef essence, with two tumblers of brandy milk punch per diem. Vital power was so completely paralyzed by depletion that no assimilation could be carried on, and the food was passed her anum with scarce a vestige of change from the condition in which it entered the mouth. Pepsine and pancreatic emulsion were freely used, with the hope of effecting artificial digestion, but to no avail.

I am induced to place this case on record for two reasons; first, that any contribution to the history of placenta praevia cannot be uninteresting to the profession in its present state of information on that point. Second, that I regard the manner of my patient's death as altogether singular.

In a future article it is my purpose to lay before the readers of this journal some ideas relative to this most terrible accident of the lying-in chamber, concerning which Professor Simpson remarks that the "peril of life is as great as though the patient were seized with yellow fever. or malignant cholera; that it is twice as dangerous as the operation of lithotomy—one in every three perishing under the former, and one in every six or eight perishing under the latter."

Every step in this direction I regard as one taken aright, since, even if finally proven to be wrong, it may still guard others from following the same path, hitherto so slightly explored.

